# How I ventilate an obese patient

**DOI:** 10.1186/s13054-019-2466-x

**Published:** 2019-05-16

**Authors:** Lorenzo Ball, Paolo Pelosi

**Affiliations:** 1 Dipartimento di Scienze Chirurgiche e Diagnostiche Integrate, Università degli Studi di Genova, Genoa, Italy; 2 Ospedale Policlinico San Martino, IRCCS per l’Oncologia e le Neuroscienze, Genoa, Italy

An increasing number of patients admitted to the intensive care unit are obese [1]. Many of them require mechanical ventilation, which may promote ventilator-induced lung injury (VILI) when applied to both injured and healthy lungs. Obesity induces functional changes in the respiratory system, resulting in a reduction of the end-expiratory lung volume, increased incidence of airway closure and formation of atelectasis, and alterations in lung and chest wall mechanics [2]. These alterations explain the high occurrence of gas exchange impairment, respiratory mechanics alterations, and hemodynamic compromise. To approach to the obese patient requiring mechanical ventilation, we propose a schematic algorithm (i-STAR, Fig. 1) as follows: (1) induction and intubation, (2) setting up initial mechanical ventilation, (3) titrating mechanical ventilation parameters, (4) assessing harmfulness of mechanical ventilation, and (5) rescue strategies.

**Fig. 1:**
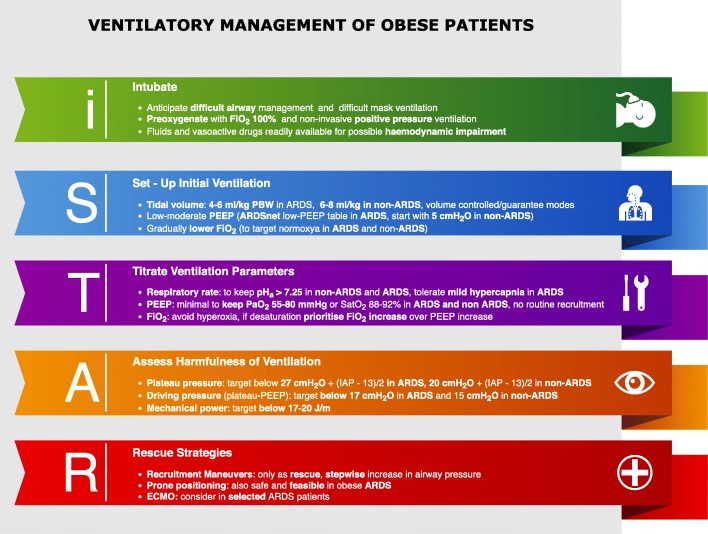
Mechanical ventilation in obese patients according to the i-STAR (Intubate, Set-up initial ventilation, Titrate ventilation parameters, Assess harmfulness of ventilation, Rescue strategies) algorithm. FiO_2_ fraction of inspired oxygen, PBW predicted body weight, ARDS acute respiratory distress syndrome, PEEP positive end-expiratory pressure, IAP intra-abdominal pressure, ECMO extracorporeal membrane oxygenation

## Induction and intubation

During induction and intubation, difficult ventilation and airway management must be anticipated, as the use of sedatives and neuromuscular blocking agents determine early loss of lung aeration and airway collapse in obese patients. We recommend the application of non-invasive positive pressure ventilation pre-oxygenation to improve gas exchange and procedural safety [3]. During the intubation phase, the fraction of inspired oxygen (FiO_2_) can be safely kept at 100% to increase the oxygen reserve. Alternative strategies including video laryngoscopes and supraglottic devices must be readily available, as well as fluids and vasoactive drugs to face hemodynamic impairment.

## Setting up initial mechanical ventilation

Once a safe airway is ensured, FiO_2_ can be lowered to avoid potentially harmful hyperoxia. Tidal volume size (*V*_T_) is a major determinant of VILI and should be titrated based on the predicted body weight (PBW) rather on the actual body weight. We recommend targeting *V*_T_ to 4–6 and 6–8 ml/kg PBW in patients with and without acute respiratory distress syndrome (ARDS), respectively, taking into account the high discrepancy between predicted and actual body weight in obese patients [4]. We prefer using volume—versus pressure-controlled mode, due to the frequent occurrence of airway closure in obese patients and observational data suggesting clinical advantages in surgical patients at high risk of developing postoperative pulmonary complications [5]. While PEEP increases end-expiratory lung volume and prevents airway collapse, it is associated with hemodynamic impairment and its optimal clinical use in obese is debated. In patients with healthy lungs, we suggest starting with a low-moderate PEEP of 5–8 cmH_2_O, while considering the ARDS Network low-PEEP table as a standard of care in obese ARDS patients [6].

## Titrating mechanical ventilation parameters

Overall, we suggest targeting gas exchange when titrating ventilation settings, as most obese patients can safely maintain PaO_2_ 55–80 mmHg and SatO_2_ 88–94% and carbon dioxide levels resulting in pHa > 7.25, also tolerating mild hypercapnia, especially in ARDS patients. We suggest changing FiO_2_ and respiratory rate as first methods to achieve these goals, respectively. However, using elevated respiratory rates may lead to increase intrinsic PEEP (PEEPi) due to airway closure and expiratory flow limitation. We strongly recommend to inspect visually the expiratory flow-time curve and to perform an expiratory hold when the presence of PEEPi is suspected. Driving pressure (∆P), i.e., the difference between plateau pressure (*P*_plat_)—PEEP, was not associated with mortality in obese ARDS patients [7]; however, this parameter has an important role in VILI and should be ideally limited to a maximum value of 17 cmH_2_O in ARDS and 15 cmH_2_O in non-ARDS obese patients. Titration of PEEP levels is controversial. Hemodynamic is more frequent than respiratory impairment in obese patients without ARDS [4]. We prefer prioritizing FiO_2_ increase over PEEP increase in patients with ARDS. Increases in PEEP should never result in an increase of ∆P, as it suggests hyperinflation and could result in worse clinical outcome [8]. However, a low-PEEP strategy might not ensure acceptable oxygenation in all patients. In patients with persistent hypoxemia, we consider using higher PEEP levels titrated on the lowest ∆P in a decremental PEEP trial [9, 10] or based on transpulmonary pressure [11]. In an observational study, higher PEEP was associated with better survival in ARDS obese patients [12], but definitive evidence is lacking, and we recommend balancing the negative effects of PEEP, especially on hemodynamics.

## Assessing harmfulness of mechanical ventilation

In obese non-ARDS and ARDS patients, *P*_plat_ should be kept below 20 cmH_2_O and 27 cmH_2_O, respectively, when clinically feasible. In obese patients, the chest wall compliance is decreased and associated with intra-abdominal pressure (IAP), estimated by bladder pressure. Therefore, we propose adjusting *P*_plat_ target based on IAP, using the following formula:

1 $$ \mathrm{Target}\ {P}_{\mathrm{plat},\kern0.75em \mathrm{adjusted}}\left(\mathrm{cm}{\mathrm{H}}_2\mathrm{O}\right)=\mathrm{target}\ {P}_{\mathrm{plat}}+\frac{\mathrm{IAP}-13\ \mathrm{cm}{\mathrm{H}}_2\mathrm{O}\ }{2} $$

Recently, the concept of mechanical power has been introduced and linked to mortality in critically ill patients [13]. This parameter can be computed as:

2 $$ \mathrm{Power}\ \left(\frac{J}{\min}\right)=0.098\bullet \mathrm{RR}\bullet {V}_{\mathrm{T}}\bullet \left({P}_{\mathrm{peak}}-\frac{\Delta  P}{2}\right) $$

where RR is the respiratory rate (min^−1^), *V*_T_ the tidal volume (L), and *P*_peak_ and ∆*P* the peak and driving pressures (cmH_2_O), respectively. Mechanical power refers to the energy transferred towards the respiratory system, and thresholds around 17–20 J/min have been proposed to minimize VILI; however, whether obese patients can tolerate higher values is unknown.

## Planning rescue strategies

We do not consider routine recruitment maneuvers as part of the standard ventilatory management of obese patients, but rather as a rescue tool in case of refractory gas exchange impairment, to be performed with gradual changes in the ventilator settings, such as stepwise increases in PEEP and/or inspiratory pressures [4]. Prone positioning has an established role as a rescue therapy in ARDS patients, and its feasibility, safety, and effectiveness have also been shown in obese patients [14]. When these conventional rescue therapies fail, extracorporeal membrane oxygenation should be considered.

The use of neuromuscular blocking agents and opioids should be limited in obese patients, in both cases preferring short-acting molecules and those with an effective antidote. Non-invasive ventilation support can be considered following extubation in selected patients [15].

In conclusion, mechanical ventilation of obese patients poses specific challenges, reflecting the profound pathophysiologic alterations frequently seen in this population. Education and training among health care professionals to improve knowledge and team working are the keys to optimize mechanical ventilation aiming at better clinical outcomes.

## Abbreviations

∆P: Driving pressure
ARDS: Acute respiratory distress syndrome
IAP: Intra-abdominal pressure
PBW: Predicted body weight
PEEP: Positive end-expiratory pressure
PEEPi: Intrinsic PEEP
*P*_peak_: Peak pressure
*P*_plat_: Plateau pressure
VILI: Ventilator-induced lung injury
*V*_T_: Tidal volume
